# The effect of Anakinra on Quality-of-Life in a family with colchicine-resistant FMF

**DOI:** 10.1186/1546-0096-13-S1-P108

**Published:** 2015-09-28

**Authors:** J van der Hilst, A Lijnen

**Affiliations:** 1 Jessa Hospital, Infectious Diseases and Immunity, Hasselt, Belgium; 2 University of Hasselt, BIOMED, Hasselt, Belgium

## Introduction

About 5-10% of patients with FMF do not to colchicine treatment to control inflammation. In these patients anti-IL1 therapy seems to be effective in controlling inflammation, although clinical trials are lacking. However, many patients on Anakinra experience side effects including headache and injection-site reactions. How Anakinra treatment influences quality-of-life (QoL) in these patients is unknown.

## Objectives

To investigate the effect of Anakinra on inflammation and QoL in a family with a severe phenotype colchicine-resistant FMF.

## Patients and methods

Four sisters of Italian descent with homozygote M694V-positive FMF, had an extraordinary phenotype with continuous inflammation, daily fevers, erysipelas-like skin lesions and serositis. Colchicine was initiated in all patient, but showed ineffective in controlling inflammation. Eventually, Anakinra was initiated. Before and during treatment with Anakinra inflammatory parameters (CRP, leukocyte-count, Serum amyloid A) were measured. One patient only started Anakinra after type AA amyloidosis had developed leading to a renal replacement therapy and renal transplantation. In the other three patients QoL questionnaire (RAND-36) was obtained, before and 4 weeks after start of Anakinra-therapy. The study was approved by the local ethical committee. Because of reimbursement was initially declined, Anakinra was discontinued. Later, it could be re-initiated. QoL questionnaires were obtained after stopping and after re-initiation of Anakinra.

## Results

During Anakinra treatment the CRP values were significantly lower than without Anakinra (mean 3.7 mg/L vs 30 mg/L, p<0.001). The QoL improved significantly after initiation of Anakinra in 5 out of 6 scales of the RAND-36 (p<0.05). The QoL deteriorated after withdrawal of Anakinra to pre-treatment level and improved again after re-initiation of Anakinra.

## Conclusion

Here we show that Anakinra can effectively suppress inflammation in a family with severe colchicine-resistant FMF. This lead to a significant improvement in quality of life.

